# The Quality of Life Among Middle-school Adolescents in Krakow

**DOI:** 10.34763/devperiodmed.20172102.124130

**Published:** 2017-08-11

**Authors:** Agnieszka Magiera, Agata Sowa, Ryszard Jacek, Agnieszka Pac

**Affiliations:** 1 Katedra Epidemiologii i Medycyny Zapobiegawczej, Wydział Lekarski, Uniwersytet Jagielloński, Collegium Medicum, Kraków, Polska

**Keywords:** adolescents, quality of life, Krakow, KIDSCREEN, sex, młodzież, jakość życia, Kraków, KIDSCREEN, płeć

## Abstract

**Aim:**

The aim of the study was to describe the quality of life of Polish adolescents living in a big city and to investigate whether there are differences in this respect between girls and boys. Moreover, we would like to compare the results concerning the quality of life of the screened adolescents from Krakow with those of their European peers.

**Material and methods:**

The survey was carried out in 2013-2015 in 17 middle schools in Krakow based on anonymous auditorium questionnaires. The analysis included the responses of 1387 pupils − 686 girls and 701 boys. In order to assess the quality of life, the Polish version of the international KIDSCREEN-27 questionnaire was used. Five dimensions of the quality of life (Qol) were analyzed. The specific dimensions of Qol were analyzed using 0-100 point scales, as well as T-scores standardized for the European population.

**Results:**

The mean values for five dimensions of quality of life assessed by the KIDSCREEN-27 questionnaire ranged from 54 pts. to 65 pts. (maximum score 100 pts.). However, the results for the Polish adolescents were lower when compared to their European peers. The greatest differences in the level of Qol between Polish adolescents and their European peers involved the following dimensions: Psychological Wellbeing and School Environment. On average, girls scored their Qol lower than boys in three out of five dimensions (Physical Well-being, Psychological Well-being, Autonomy & Parents; p<0.001). Additionally, using the norm data for the Polish population (sex and age specific), more girls than boys were classified as having low Qol regarding the School Environment (23.5%vs 14.8%; p<0.001).

**Conclusions:**

Polish adolescents scored their Qol lower than their European peers. The quality of life for girls was significantly lower than of boys, except for the relation with their friends and peers (Social Support & Peers).

## Streszczenie

**Cel:**

Celem pracy była ocena jakości życia adolescentów zamieszkujących w dużym mieście oraz ocena różnic w jakości życia pomiędzy dziewczętami i chłopcami. Ponadto, porównano wyniki jakości życia badanych adolescentów z wynikami uzyskanymi przez ich europejskich rówieśników.

**Materiał i metody:**

Badanie zrealizowano w latach 2013-2015 w 17 gimnazjach w Krakowie w oparciu o anonimowe ankiety audytoryjne. Do analizy włączono 1387 uczniów – 686 dziewcząt i 701 chłopców. Do oceny jakości życia wykorzystano polską wersję międzynarodowego kwestionariusza do badania jakości życia dzieci i młodzieży KIDSCREEN-27. Analizowano 5 wymiarów jakości życia. Dla poszczególnych wymiarów jakości życia uwzględniono w analizie wyniki surowe przeliczone na skalę od 0 -100 pkt oraz standaryzowane na populację europejską (T-score).

**Wyniki:**

Średnia jakość życia młodzieży oceniona kwestionariuszem KIDSCREEN-27 mieściła się w przedziale 54 pkt − 65 pkt (maksymalny wynik 100 pkt), jednak wyniki te były niższe niż ich rówieśników z populacji europejskiej. Największe różnice w poziomie jakości życia pomiędzy badaną młodzieżą, a ich europejskimi rówieśnikami dotyczyły wymiarów: samopoczucie psychiczne i środowisko szkolne. Przeciętnie dziewczęta niżej oceniały swoją jakość życia niż chłopcy w 3 z 5 wymiarów (samopoczucie fizyczne, samopoczucie psychiczne niezależność i rodzice; p<0,001). Dodatkowo, po odniesieniu do norm polskich dla płci i wieku, również znacząco więcej dziewcząt niż chłopców miało niską jakość życia w wymiarze dotyczącym środowiska szkolnego (23,5% vs 14,8%; p<0,001).

**Wnioski:**

Badana młodzież gimnazjalna niżej oceniała swoją jakość życia w porównaniu do ich europejskich rówieśników. Jakość życia badanych dziewcząt była znacząco niższa niż chłopców z wyjątkiem oceny dotyczącej relacji z przyjaciółmi i rówieśnikami.

## Background

According to the World Health Organization (WHO), quality of life (Qol) is defined as *“perception by the entity’s position in life, in the context of culture and value systems in which they live and the relation to its objectives, expectations, standards and interests* “[1]. The term “quality of life” in relation to both individuals and population groups is very important from the point of view of identifying diverse needs and proposing preventive measures which are best suited for these population groups.

Adolescents belong to a population group that is considered to be more sensitive (vulnerable) or susceptible to adverse situations and tendencies of its development [2]. The quality of life in adolescence poses a particular challenge for the researcher, as the period of adolescence is the time when significant changes are observed; physical and mental, as well as intellectual, social, and emotional individual development is very intense [3]. The quality of life of adolescents should cover the most important dimensions for this age group and most important problems young people are facing. Feeling good, being satisfied with oneself and having an overall positive attitude, as well as a positive self-image, good friends and good family relations are very important [4]. Friends and peers seem to be the most significant factor for young people.

It is important to feel self-esteem and cope with negative emotional states – fear and loneliness. Social skills, especially establishing and maintaining contacts and close relations with peers and adults are also vital. Issues of identity gain particular importance in adolescence. Therefore, for the quality of life of adolescents it may be very important to develop their own identity and achieve independence. The structure of the quality of life of children and adolescents indirectly derives from the conditions which are generally associated with this stage of development [5].

The KIDSCREEN European project developed a standardized quality of life questionnaire for children and adolescents aged 8-18 with normative norms collected across 13 countries in Europe [6]. The KIDSCREEN project deals with the international aspect of HRQL by developing questionnaires in ten parallel language versions; these could be applied in population studies of persons considered healthy, as well as in clinical studies [7]. This questionnaire was used in different populations, for example among very preterm-born adolescents [8], among deaf children and adolescents [9] or adolescents with mental disorders [10].

In Poland there are only a few studies describing the quality of life of adolescents, mainly concerning youth with somatic or psychological disabilities. Studies on the quality of life of adolescents in Poland were carried out less frequently and usually they are part of international research projects. Examples of recent studies carried out in Poland among children and adolescents aged 8-19 are: project ADOPOLNOR [11], the periodical HBSC survey [12], or the Children’s Worlds Survey [13]. None of these studies had been conducted in Krakow and the Malopolska Region. There is much more research on adolescents’ quality of life in the international literature [4, 6, 13, 14, 15, 16, 17, 18, 19, 20, 21]. These studies are using different methods/ scales to assess QoL and dealing with its different dimensions. Generally, research showed that the quality of life is higher among boys than girls [14, 15, 16, 17, 18, 19, 20, 21, 22] and that among the different dimensions of Qol studied, the lowest score was observed for adolescents’ psychological well-being, the school environment and relations with parents [14, 15].

The aim of the study was to describe the quality of life of Polish adolescents living in a big city and to investigate whether there are differences in the quality of life between Polish girls and boys. Moreover, we would like to compare the results of the quality of life of the screened adolescents from Krakow with the results for their European peers.

## Material and methods

### Ethics

The study was reviewed and approved by the Jagiellonian University Bioethics Committee (approval number: KBET/ 143/B/2013). During the recruitment phase of the study, school principals of all the participating schools gave their approval for the study. Moreover, individual written informed consent was obtained from the parents of the participating schoolchildren.

### Sample and data collection

A cross-sectional study was conducted among schoolchildren from 17 middle schools from different districts of Krakow between December 2013 and February 2015. Information about the study and its aims were given to the schoolchildren’s parents during periodical teacher-parent meetings. Only 70% of the parents expressed their opinion and gave feedback about their child’s participation (either approval or refusal) in the study. Among those who responded, 14% refused to agree to their child’s participation in the study. Finally, data from 1465 adolescents were collected, that is from 90.2% of those who agreed to participate.

The questionnaire, which took approximately 10-15 minutes to fill out, was administered in school classrooms and filled out individually by the adolescents in the presence of the research assistant in class to provide assistance when needed.

This analysis was performed for 1387 adolescents, 78 questionnaires were excluded, because of missing data for the KIDSCREEN-27 questionnaire. The sample consisted of 430 adolescents from the 1st (31%), 490 from 2nd (35.4 %) and 467 from 3rd grades (33.7%). The average age was 14.6 years (SD 0.97). The sample included 686 (49.3%) girls and 701 (50.7%) boys.

### Measurement

The quality of life was measured using the Polish version of the KIDSCREEN-27 questionnaire [7]. KIDSCREEN is an international, validated instrument for cross-cultural comparisons. The 27-item, child version was used in this study. It covers 5 dimensions:

1. *Physical Well-Being* (5 items): this explores the level of physical activity, energy and fitness of the child/ adolescent.
2. *Psychological Well-Being* (7 items): this involves psychological well-being, such as positive emotions, satisfaction with life and the absence of loneliness and sadness.
3. *Parent Relations and Autonomy* (7 items): this involves the bond with parents and atmosphere at home and the extent to which the child/ adolescent feels loved and supported by the family.
4. *Social Support and Peers* (4 items): this explores the quality of the child’s/ adolescents’ social relations and interactions with friends and peers.
5. *School Environment* (4 items): this determines the child’s cognitive ability, the ability to learn. It makes it possible to assess feelings towards school and relationships with teachers [6].

The response range for each KIDSCREEN-27 item is based on a 5-point Likert scale. The scale indicates the frequency of certain behaviors or feelings (1=never to 5=always) or the intensity of an attitude (1=not at all to 5=extremely). The time frame refers to the previous week [6]. The original KIDSCREEN instrument, as well as the Polish version of this instrument were validated [7]. The Polish version of the child KIDSCREEN-27 has shown satisfactory validity, as well as reliability to be used in the Polish population [7].

The raw scores on particular KIDSCREEN-27 subscales have been transformed to a range of 0-100. These values allow for the interpretation of QoL across each dimension by age and gender, and represent their overall mean QoL across each individual dimension.

To compare the results of our sample with those of the reference population from the KIDSCREEN project, the scores were transformed into T-scores with a mean of 50 and a standard deviation (SD) of 10 based on the guidelines developed by the authors of the scale for the international sample of adolescents aged 8-18 years old [6].

We have also decided to use Polish norm data to assess the level of quality of life. Each participant was assessed to have low, average or high quality of life as compared to the age and gender norms for the Polish population as described in the paper by Mazur et al. [7]. Low Qol was defined as a score lower than mean minus one standard deviation for sex and age normative data (transformed into 1-100 pts.), while high Qol was defined as higher than mean plus one standard deviation. Polish normative data were used to account for country-specific differences [7]. The KIDSCREEN manual groups individuals’ scores into high and low categories which specify the range of each KIDSCREEN dimension by its extreme ends. In order to carry out a detailed further examination of the data, it was decided to include a middle-range score.

### Statistical analyses

Descriptive statistics for the variables of age, sex and quality of life are presented as means (M) and standard deviations (SDs), or as percentages. In order to investigate differences between boys and girls in relation to KIDSCREEN-27 dimensions, the t-test or Mann-Whitney test were used for normal-distributed and non-normal distributed scores, respectively.

All the analyses were conducted using IBM SPSS Statistics 24, with statistical significance set at p<0.05.

## Results

Based on data from 686 adolescent girls and 701 boys we have observed that Quality of life in all the dimensions was scored over 50 pts. (on the scale 0-100 pts.), but there were variations across the different dimensions. The lowest quality of life was observed in relation to School Environment, with the mean value of 53.9 pts. There were no differences between boys and girls in their assessment of this dimension. All the other Qol dimensions examined were scored (in the whole group) at a similar level. However, differences were observed between boys and girls in relation to Physical Well-being, Psychological Well-being and Autonomy & Parents, with boys scoring much higher than girls on their quality of life in these dimensions. The detailed data were presented in table I.

**Table I:** The quality of life of adolescents in Krakow in different KIDSCREEN-27 dimensions (on the scale of 0-100 pts.). Tabela I. Jakość życia badanej młodzieży w różnych wymiarach kwestionariusza KIDSCREEN-27 (na skali 0-100).

KIDSCREEN-27 dimension Wymiar KIDSCREEN-27	Total *Ogółem* N=1387		Girls *Dziewczęta* N=686		Boys *Chłopcy* N=701		p
	Mean *Średnia*	SD	Mean *Średnia*	SD	Mean *Średnia*	SD	
Physical Well-being *Samopoczucie fizyczne*	63.38	18.84	58.48	18.64	68.18	17.78	<0.001
Psychological Well-being *Samopoczucie* *psychiczne*	63.83	20.41	58.44	21.37	69.10	17.93	<0.001
Autonomy & Parents *Niezależność i Rodzice*	63.72	20.48	59.21	21.22	68.13	18.71	<0.001
Social Support & Peers Wsparcie społeczne i rówieśnicy	64.81	21.37	65.57	22.13	64.06	20.59	0.091
School Environment *Środowisko szkolne*	53.89	19.68	53.44	19.89	54.34	19.47	0.351

We also wanted to compare the quality of life of Polish adolescents with their peers from other countries, so we used data transformation into T-score values based on the original KIDSCREEN project with international norm data developed for a reference European population aged 8-18 years. Based on this analysis, in our sample we have observed lower T-scores than the average KIDSCREEN score (mean 50) in all of the dimensions (table II). Particularly low scores were observed for the Psychological Well-being of adolescents (mean: 42.77) and School Environment (mean: 42.91) – these results were much lower than in the European population and even lower than the 25^th^ percentile. The above dimensions were also scored lower than in the study of Polish adolescents in 2003 [6]. The highest Qol was observed in the Physical Well-being dimension (mean: 46.20), however it was still lower than the results for both Polish and European populations screened in 2003 (means: 49.04 and 50, respectively). For the other two dimensions of Qol: Autonomy & Parents and Social Support & Peers, our results were similar to those observed earlier [6] in the Polish population of adolescents and lower than those reported for the European reference population. The details are presented in table II.

**Table II:** Comparison of the quality of life of adolescents in Krakow to international standards from the KIDSCREEN project (T-score). Tabela II. Porównanie jakości życia badanej młodzieży krakowskiej w odniesieniu do wartości normalizowanych w projekcie KIDSCREEN (T-score).

KIDSCREEN-27 dimension *Wymiar KIDSCREEN-27*	Krakow 2013-2015 *Kraków 2013-2015*	Poland 2003 *Polska 2003*	European norm data for ages 8-18 2003 *Normy europejskie 8-18 lat 2003*			
	M±SD*	M±SD*	M±SD*	25 centile *25 centyl*	50 centile *50 centyl*	75 centile *75 centyl*
1. Physical Well-being *Samopoczucie fizyczne*	46.20±9.15	49.04±9.26	50±10	42.53	49.63	55.60
2. *Samopoczucie* Psychological *psychiczne* Well-being	42.77±9.47	46.55±8.97	50±10	43.21	48.45	55.96
3. *Niezależność* Autonomy & *i* Parents *rodzice*	44.86±9.43	44.99±8.28	50±10	42.86	49.47	55.75
4. Social Support & Peers *Wsparcie społeczne i rówieśnicy*	44.35±9.91	45.05±9.49	50±10	44.40	49.79	57.83
5. School Environment *Środowisko szkolne*	42.91±8.18	46.18±9.01	50±10	42.94	48.09	54.40

*M − Mean/*średnia*; SD – Standard Deviation/*odchylenie standardowe*

**Fig. 1:**
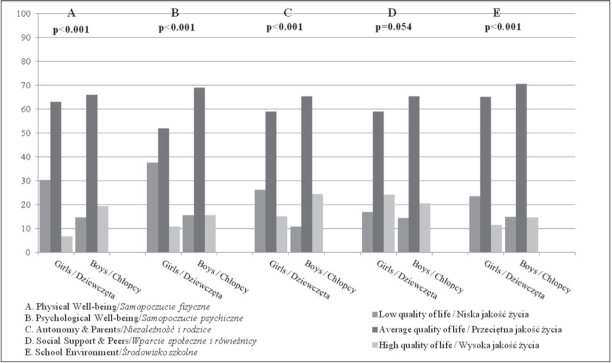
Classification of the level of quality of life adolescents in Krakow by sex − external criteria for classification from paper [7] (A − Physical Well-being; B – Psychological Well-being; C – Autonomy & Parents; D – Social Support & Peers; E – School Environment). Ryc. 1. Skategoryzowany poziom jakości życia młodzieży krakowskiej wg płci − kryteria zewnętrzne podziału z pracy [7] (A – Samopoczucie fizyczne; B – Samopoczucie psychiczne; C – Niezależność i rodzice; D – Wsparcie społeczne i rówieśnicy; E – Środowisko szkolne).

Furthermore, participants across the five KIDSCREEN-27 dimensions were grouped into low, middle and high Qol based on the Polish norm ranges calculated in respect to the sex and age of the participant. Figure 1 shows the comparison between girls and boys. Significant differences were observed in four KIDSCREEN-27 dimensions: Physical Well-being, Psychological Well-being, Autonomy & Parents and School Environment. In the Physical Wellbeing dimension, high QoL was found in only 6.7% of girls and 19.4% of boys and low quality of life for 30.3% girls and 14.6% boys. The highest prevalence of low Qol was observed for Psychological Well-being – 37.5% of girls and 15.4% of boys followed by the Autonomy & Parents dimension (26.1% and 10.6%, respectively) and School Environment (23.5% vs 14.8%). The quality of life related to contacts with friends and peers (Social Support & Peers) was similar for girls and boys with over 20% of the respondents classified as having good Qol (fig.1).

## Discussion

Our study showed that Polish adolescents living in a big city scored their quality of life as average (mean scores for the five dimensions from 54 pts. to 65 pts.). However, this assessment was lower than their peers who were screened in the KIDSCREEN project [6].

Girls reported lower quality of life than boys in three out of five KIDSCREEN-27 dimensions. In the present study, girls scored significantly lower on Physical Well-being and Psychological Well-being and Autonomy & Parents, compared to boys. In addition, when we accounted for the age of the screened adolescents, more girls than boys were classified as having a low quality of life. What is more, this observation was confirmed in other studies, both in the Polish population, as well as in different countries [6, 7, 11, 12, 13, 14, 15, 16, 17, 18, 19, 20, 21, 22]. In the Polish study “Health and Well-Being of Adolescents in Wielkopolska Province” using the Polish version of the Youth Quality of Life questionnaire, more boys than girls were very happy with their lives, and fewer girls expressed satisfaction with themselves [11]. Also studies using the KIDSCREEN-52 instrument showed that boys scored higher than girls on the dimensions of: Physical Well-being, Psychological Well-being, moods and emotions, self-perception and autonomy [16]. Gaspar et al. report that boys present higher scores than girls, except in the dimension described School Environment [17]. On the other hand, the study carried out in Greece showed that girls had a Significantly higher quality of life in the dimension of School Environment as compared to boys [18].

Other authors also indicate that there are gender differences regarding the quality of life in longitudinal studies. Boys reported a significantly higher quality of life than girls in three dimensions: Physical Well-being, Psychological Well-being and Autonomy and Parent Relations [19]. Similar results were observed in Estonian and Spanish cohort observations [15, 20].

Lower quality of life in adolescent girls might be explained by biological differences and differences in gender roles and gender socialization. Biological sex differences such as earlier puberty and brain development can underpin explanations of lower quality of life in girls. Gender roles may contribute to lower physical and psychological well-being in girls [21].

The mean scores (54 pts. – 65 pts.) achieved by girls and boys on the scale of 0-100 points were slightly above the middle of the scale, however, compared to the reference population studied, our adolescents received lower scores than their peers. Michel et al. referred to the UNICEF report which provides a comparative assessment analysing children of 21 European countries, including Poland, regarding six aspects of child well-being. Poland was placed at the bottom of the table, with the lowest child well-being reported [22]. Furthermore, in the OECD report “How’s Life in Poland?” the authors indicated that the life satisfaction of Polish children is among the lowest amongst OECD countries [23]. The differences between countries can be related to lifestyle, upbringing in Poland, norms of behavior, Polish mentality. Therefore, differences in the quality of life between countries point to the importance of the national context for the health and well-being of adolescents [22].

### Strengths and limitations of the present study

The Quality of life of adolescents was assessed using the KIDSCREEN-27 questionnaire prepared as a multinational instrument and validated both internationally and in Poland. Use of the KIDSCREEN instrument allows cross-cultural comparisons, as well as country-specific analysis, because of normative data prepared for international and Polish populations. In addition, the sample size was big enough to receive reliable estimates. This study provides information about the quality of life of the middle-school adolescent population and confirms gender differences. It is the first of this kind in Krakow and the Malopolska region.

Some limitations should also be mentioned. We decided to study adolescents at schools and only about 60% of the pupils responded to the questionnaire, because of the lack of their parents’ informed consent. Information about the study was given to the parent during periodical teacher-parent meetings, however we did not receive any information (either approval or refusal to participate) for 30% of the children. This might later result in a slight overestimation of our results. Another limitation is the lack of socioeconomic data regarding the participants. Furthermore, the comparison of our adolescent population and their European peers may be distorted due to different variables, for example the change of quality of life over time, socioeconomic data, living in a big city.

## Conclusions

Summing up, secondary-school pupils from Krakow scored their quality of life as average (54 pts.-65 pts.). However, their Qol results compared to international norms showed that this assessment was lower than those observed in other European countries. Girls reported lower quality of life than boys did in most of the Qol dimensions analyzed – only relations with friends and peers were similar for both groups. The lowest scores were observed in quality of life related to the school environment.
